# Utility of MRI as the primary imaging tool in hypertension

**DOI:** 10.1186/1532-429X-16-S1-P248

**Published:** 2014-01-16

**Authors:** Amy E Burchell, Laura E Ratcliffe, Andreas Baumbach, Angus K Nightingale, Nathan Manghat

**Affiliations:** 1 Cardiology, Bristol Heart Institute, Bristol, UK; 2 School of Clinical Sciences, University of Bristol, Bristol, UK; 3 Physiology and Pharmacology, University of Bristol, Bristol, UK; 4 NIHR Biomedical Research Unit, University Hospitals Bristol NHS Foundation Trust, Bristol, UK

## Background

In patients with early onset or drug resistant hypertension (HTN) exclusion of secondary causes with imaging and endocrine testing is recommended. Traditionally a combination of echocardiography, renal ultrasound and renal CT angiography are used to investigate patients. Magnetic resonance imaging (MRI) is the gold standard imaging modality for the quantification of cardiac volumes and masses, with the benefit of being non-invasive and non-ionising. MRI can sensitively screen for renal artery stenosis and accurately evaluate renal artery anatomy. This is of particular relevance in the age of interventional therapies for resistant hypertension such as renal denervation. We proposed that a single MRI visit could provide all the imaging required in the routine evaluation of patients with HTN.

## Methods

Patients attending a specialist hypertension clinic with early onset (<40 years), drug resistant or challenging drug intolerant HTN underwent a Hypertension Protocol MRI scan assessing for secondary causes and target organ damage. This included a full cardiac MRI with late gadolinium enhancement, imaging of the kidneys and adrenals, and MR angiography (MRA) of the renal arteries, aorta and cerebral vessels. Data is presented as mean ± SD.

## Results

**Figure 1:**
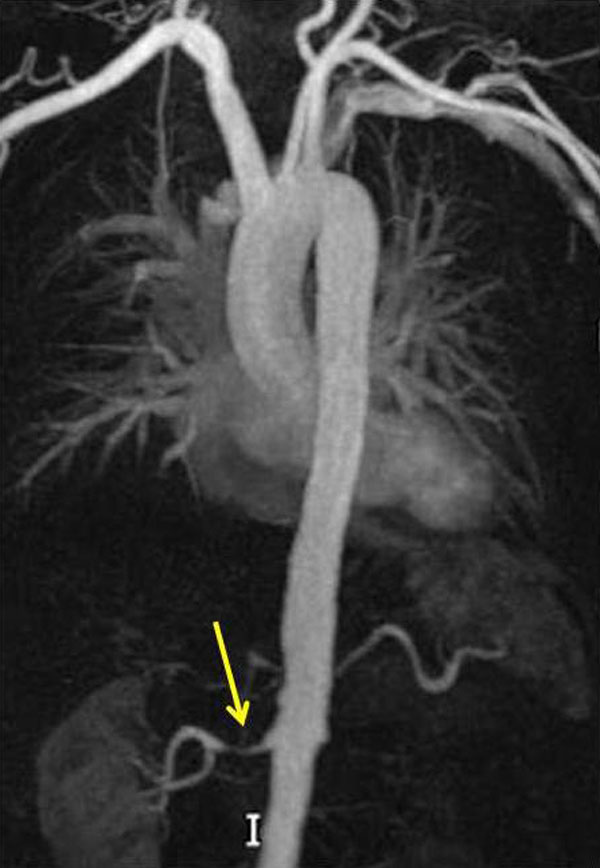
**Right renal artery stenosis**.

71 patients (36 male), aged 54 ± 15 years, had an office blood pressure of 177 ± 28/98 ± 16 mmHg on 3.6 ± 2 (range 0-8) antihypertensive medications. 78% of patients had left ventricular hypertrophy by left ventricular mass index (94 ± 26 g/m2); other pathological findings are summarised in Table 1. 21% had ≥1 accessory renal artery with 80% of patients anatomically suitable for renal denervation by current European guidelines. Figure 1 shows an example of renal artery stenosis on an MRA reconstruction.

**Table 1:** Clinically relevant findings on the Hypertension Protocol MRI scans for this hypertensive cohort.

Possible secondary causes of hypertension	No. of patients (n = 71)	Prevalence (%)
Nil	33	46

Obesity alone	27	38

Renal artery stenosis	5	7

Adrenal mass	4	6

Single hypoplastic kidney (renal coloboma synd.)	1	1

Multinodular goitre (normal TSH)	1	1

**Target organ damage**		

Left ventricular hypertrophy	55	78

Myocardial infarction	8	11

Aortic dilatation	6	9

Cerebral microaneurysm	4	6

Splenic artery aneurysm	1	1

Coeliac axis stenosis	1	1

## Conclusions

MRI is a safe and effective method of screening for secondary causes of HTN and could replace the combination of echocardiography, renal ultrasound and CT imaging. MRI could offer novel imaging strategies for risk stratifying patients with HTN via assessment of aortic distensibility, pulse wave velocity and cerebral blood flow. It provides additional information for patients being considered for renal denervation and is likely to be a cost effective first line investigation for patients with possible secondary causes of hypertension.

## Funding

Scans were performed as part routine assessment through an NHS funded Specialist Hypertension Clinic with additional support from the Bristol NIHR Biomedical Research Unit. Dr Burchell is funded by a University Hospitals Bristol NHS Trust Research Fellowship.

